# Glowing Green: A Quantitative Analysis of Photoluminescence in Six North American Bat Species

**DOI:** 10.1002/ece3.71885

**Published:** 2025-07-28

**Authors:** Briana J. Roberson, Santiago Perea, Daniel DeRose‐Broeckert, Steven B. Castleberry

**Affiliations:** ^1^ D.B. Warnell School of Forestry and Natural Resources University of Georgia Athens Georgia USA; ^2^ The Nelson Institute for Environmental Studies & The Department of Forest and Wildlife Ecology University of Wisconsin‐Madison Madison Wisconsin USA

**Keywords:** adaptive significance, bats, museum specimens, photoluminescence

## Abstract

Photoluminescence produced by excitation with ultraviolet light has been documented in an increasing number of nocturnal–crepuscular mammal species. Here, we provide a quantitative analysis to confirm visual observations of UV‐induced photoluminescence in six North American bat species. We used museum specimens to examine wavelength at peak photoluminescent emission, within and among species. We observed green photoluminescence on the wings, uropatagium, and hind limbs of all 60 museum specimens examined. Spectral scans revealed a consistent emission peak between 520 and 552 nm corresponding to the observed green color. We found no differences in wavelength between species or sexes. Wavelength was not related to specimen age, supporting the use of museum specimens for detection of photoluminescence. Our results suggest the potential for photoluminescence to be homologous in origin among the species we examined. We emphasize the need for further exploration into potential evolutionary and functional roles of photoluminescence across mammalian taxa.

## Graphical abstract

We provide a quantitative analysis to confirm visual observations of UV‐induced photoluminescence in six North American bat species. We observed green photoluminescence on the wings, uropatagium, and hind limbs of all 60 museum specimens examined. Our results suggest the potential for photoluminescence to be homologous in origin among the species we examined.

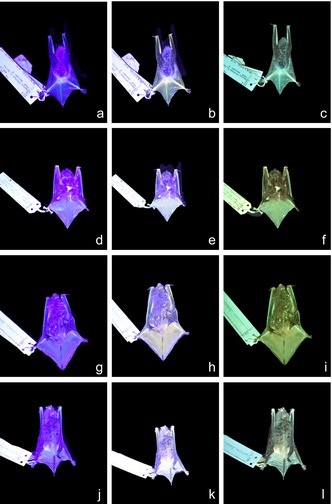

## 1 Introduction

Photoluminescence describes the phenomenon by which molecules absorb photons at a shorter wavelength and produce emission of light at a longer wavelength. The term 'photoluminescence’ encompasses several mechanisms, including fluorescence, phosphorescence, and light scattering (Shinde et al. 2012; Reinhold 2023). The phenomenon of photoluminescence in biological systems is historically well documented in plants, invertebrates, and marine organisms (Lagorio et al. 2015; Poding et al. 2024). However, mammals have recently garnered attention for displaying photoluminescence across many taxa (Tumlison and Tumlison 2021; Reinhold et al. 2023; Travouillon et al. 2023).

There is discourse as to whether ultraviolet‐induced mammal photoluminescence has adaptive significance that would explain its widespread existence. The trait is currently thought to be attributed to adaptation in low‐light conditions, as nearly all species currently confirmed to photoluminesce are nocturnal, crepuscular, or fossorial (Kohler et al. 2019; Anich et al. 2021; Pynne et al. 2021). Most theories can be grouped into potential advantages for predator evasion, communication, or improving vision in low‐light conditions (Kohler et al. 2019; Anich et al. 2021; Olson et al. 2021; Pynne et al. 2021). In addition, most light available during nocturnal and crepuscular hours is within the ultraviolet and blue range (Endler 1993; Cohen et al. 2014). The presence of visible light through ultraviolet‐induced photoluminescence may provide adaptive advantages such as enhancing visual signaling in nocturnal or crepuscular species (Johnsen et al. 2006). However, there is also the possibility that photoluminescence serves no explicit ecological function. Marshall and Johnsen (2017) provide a set of guidelines to evaluate the potential ecological relevance of photoluminescence, which include realistic conditions for photoluminescence excitation and visual detection, and behavioral modification in the presence of photoluminescence. No mammal photoluminescence is currently confirmed to satisfy all requirements to infer ecological function, and Reinhold et al. (2024) found no evidence for the visual function of blue photoluminescence in Australian ground‐dwelling mammals based on these guidelines. Evaluating observed characteristics of photoluminescence with these stipulations in mind is important when hypothesizing potential functions.

Bats (order Chiroptera) are an interesting model to study photoluminescence due to their distinct morphology, adaptations for sensory perception, and social systems. Photoluminescence has been documented in several bat species, including presence on the foot bristles of Mexican free‐tailed bats ( *Tadarida brasiliensis mexicana* ; Gual‐Suárez et al. 2024) and the wings of eastern tube‐nosed fruit bats ( *Nyctimene robinsoni* ; Reinhold 2022). In addition, Greater Antillean long‐tongued bats ( *Monophyllus redmani* ) exhibit piebald spots that are enhanced visibly in the presence of ultraviolet light (Kurta et al. 2023). Ultraviolet light and its byproducts within the visual spectrum may play a significant role in bat behavior and communication, but further information is needed to support the potential for photoluminescence to serve an explicit function in bats.

Patterns between natural history or phylogeny and characteristics of fluorescence in mammals are not easily isolated (Reinhold 2023), especially because many studies have described fluorescence qualitatively by perceived color or intensity (Pine et al. 1985; Kohler et al. 2019; Pynne et al. 2021; Tumlinson and Tumlinson 2021; Reinhold et al. 2023). Several studies have quantified spectra and noted differences in intensity of emission (Anich et al. 2021) or performed in‐depth analyses relating characteristics of emission to life history (Travouillon et al. 2023). Examining quantitative differences in the characteristics of photoluminescent emission among closely related species can provide support for potential phylogenetic patterns and additional information to evaluate ecological significance. If closely related species exhibit similar characteristics of emission, it can be inferred that there is a shared physiological mechanism producing luminescence. In addition, documentation of wavelengths of photoluminescence can be used to evaluate potential for visual detection.

Here, we provide a quantitative analysis to confirm visual observations of UV‐induced photoluminescence in six species of bats native to North America. To do so, we examined spectral characteristics of emission for differences within and among species and interpreted the results to offer hypotheses for the evolution and function of photoluminescence in bats. We hypothesized that the peak wavelength of emission and therefore the proximate origin would be homologous among the species examined.

## 2 Materials and Methods

### 2.1 Specimens

We examined female and male adult museum specimens of big brown bats ( *Eptesicus fuscus* ), eastern red bats ( *Lasiurus borealis* ), Seminole bats ( *Lasiurus seminolus* ), southeastern myotis ( *Myotis austroriparius* ), gray bats ( *Myotis grisescens* ), and Brazilian free‐tailed bats ( *Tadarida brasiliensis* ) from the Georgia Museum of Natural History (GMNH; Athens, Georgia, USA) mammal collections. Most specimens were collected in Georgia, USA, but collection localities ranged geographically across the United States, including South Carolina, Tennessee, Illinois, and California. Specimens ranged from 22 to 103 years since collection (Table S1).

We first visibly observed specimens for the presence of photoluminescence under 410 nm ultraviolet light with an emission spectrum ranging from 395 nm to 425 nm (±15 nm full width half maximum) using a yellow UV‐filtering lens (Circus, New York, USA, and LPSAFP, Amazon, China) to reduce visual noise from UV and blue light. We photographed specimens in a dark box using a Nikon D5600 camera with an AF‐P NIKKOR 18–55 mm lens (Nikon, Melville, New York, USA). For comparison, photographs were taken under only UV light and through the UV‐filtering lens. In addition, we used a MIDOPT LP470 yellow longpass filter (Midwest Optical Systems Inc., Palatine, Illinois, USA) to completely filter out incident light under 470 nm and photograph photoluminescent emission color objectively without influence from ultraviolet and blue wavelengths. Specimen photos were digitized with a black background for visualization.

### 2.2 Measurements

We quantified specimen photoluminescent emission spectra with an ILT950 spectroradiometer (International Light Technologies, Peabody, Massachusetts, USA), which has a wavelength accuracy of ±0.5 nm, using an integration time of 4000 ms. The probe was positioned 10 mm over the ventral surface of the uropatagium, which was the body region with the greatest area of visually observed photoluminescence available to scan. Intensity of light was recorded as irradiance (μW/cm^2^). To account for confounding light in the dark room and to isolate photoluminescent emission from the UV light, we recorded initial scans with no light present and reference scans of the UV light before specimen scans, using an A253 diffuse reflectance standard reference panel (International Light Technologies, Peabody, Massachusetts, USA). We scanned each specimen once and subtracted dark scans and reference scans from specimen scans using the following formula to ensure analyses only included light produced by specimens.

$$\begin{array}{c} \text{Photoluminescence}=|\;\text{specimen irradiance values}\\ -\left(\text{dark scan irradiance values}+\text{reference irradiance values}\right)| \end{array}$$

We then processed scans to identify the wavelength value at the emission peak for each specimen. We assessed variation in mechanical peak wavelength detection by calculating the standard deviation of peak wavelength values from 5 duplicate scans taken from the same location on a single specimen.

### 2.3 Statistical Analysis

All analyses, scans, and data processing were conducted in R version 4.4.1 (R Core Team 2023). We examined specimen emission spectra for inter‐and intraspecific (between sexes) differences in wavelength (nm) at the emission peak (hereafter ‘peak wavelength’). We performed a Shapiro–Wilk test for normality and found that response variables were not normally distributed. Therefore, we used Kruskal–Wallis non‐parametric tests to determine if peak wavelength differed among species and between males and females.

To determine if specimen age influenced scan results, we compared a model with specimen age since collection as a predictor variable to a null model with only an intercept term (no trend term) with generalized linear models (GLMs) using the stats R package. We initially conducted the analysis for each species and detected no differences. Therefore, we combined all specimens examined for the final analysis. We used a gamma distribution because the response variable was continuous and not normally distributed. Models were compared using Akaike's information criterion (AIC) with a threshold of ≥ 2 units to determine if the age‐predictor model differed in fit from the null model (Burnham and Anderson 2003).

## 3 Results

### 3.1 Visual Observations

**FIGURE 1:**
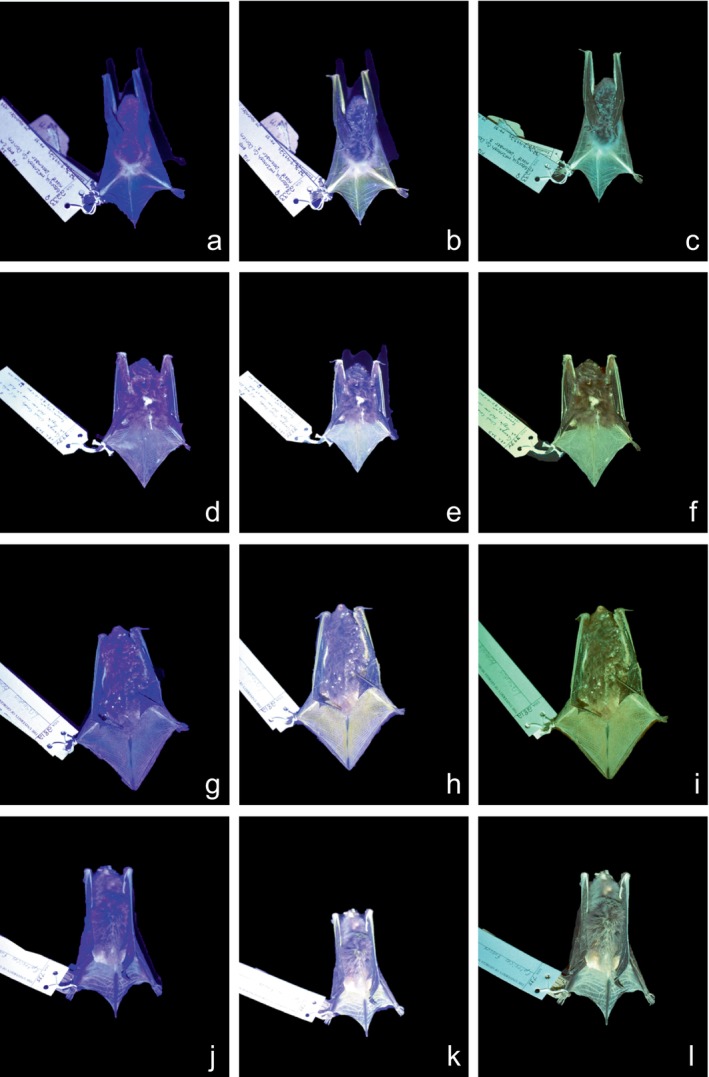
Specimens of *Myotis austroriparius* (a–c), *Lasiurus seminolus* (d–f), *Lasiurus borealis* (g–i), and *Eptesicus fuscus* (j–l). Specimens were illuminated under 410 nm light and photographed under UV light alone (column 1), under filtration using yellow‐tinted UV‐filtering lens (column 2), and under filtration using a 470 nm longpass filter (column 3).

Bright green photoluminescence was observed on the uropatagium, wings, and hind limbs of all 60 specimens examined (Figure 1). While differences in photoluminescence intensity were observed, color was consistently observed to be green among all specimens. The use of a UV‐filtering lens for visual observations was critical in observing true emission color by mitigating the strength of UV and blue light.

### 3.2 Spectral Characteristics

**FIGURE 2:**
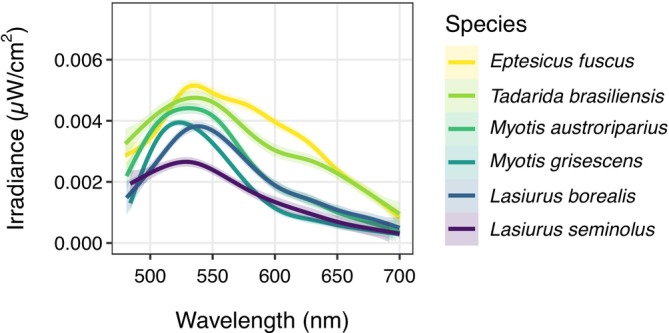
Peak wavelength of photoluminescence emission spectra for museum specimens of six North American bat species (*n* = 10 for each species). Shaded regions represent 95% confidence intervals.

After scan processing, we identified a peak in emission wavelength ranging from 520 to 552 nm, consistent with the observed green color (Figure 2). The standard deviation of mechanical peak wavelength detection was ±2.5 nm. We found no interspecific differences in peak wavelength (Kruskal–Wallis: *χ* ^2^ = 10.595, df = 5, *p* > 0.05). Because there was no difference in peak wavelength among species, we pooled species and found no differences between sexes (Kruskal–Wallis: *χ* ^2^ = 0.803, df = 1, *p* = 0.37). Specimen age did not influence wavelength values (AIC GLM = 412.242, AIC null model = 411.646).

## 4 Discussion

We provide quantitative observations of UV‐induced photoluminescence in six North American bat species. These observations contribute to current findings of photoluminescence being widespread across mammal taxa (Tumlinson and Tumlinson 2021; Reinhold et al. 2023; Travouillon et al. 2023). Our observations are consistent with similar yellow‐green photoluminescence on the ventral wing surfaces of *Nyctimene robinsoni* in Australia (Reinhold 2022) but are the first confirmation of similar green patagial photoluminescence in North American bat species. Our observations differ from the red UV‐fluorescence found across distantly related mammal taxa that can be attributed endogenously to porphyrins (Toussaint et al. 2023; Olson et al. 2021) and other colors of pelage fluorescence in mammals currently thought to originate from tryptophan metabolites (Nicholls and Rienits 1971; Reinhold 2023).

Our results provide additional information needed to assess evolutionary drivers of photoluminescence in the species examined. Because we found no link between peak wavelength and sex, our results do not provide evidence to support sexual selection as a mechanism influencing the color of photoluminescence. In addition, because there were no interspecific differences in peak wavelength, we infer similarity in the proximal source of photoluminescent color through shared luminophores and therefore suggest the trait is a synapomorphy among the species examined. Consequently, we can infer that if color is the primary determinant of an ecological function of photoluminescence, the function would be shared among the species examined. These species differ in their roosting ecology, both by environmental preference and social behavior. Species in the genus *Lasiurus* are foliage roosters; therefore, emission spectra of photoluminescence would be expected to be similar to chlorophyll‐a fluorescence with a peak at 680 nm (Lagorio et al. 2015) if functioning to provide camouflage among foliage. Species in the genus *Myotis*, *T. brasilensis* , and *E. fuscus* primarily roost in structures, including cavities, caves, and hollow trees or under bark (Davis et al. 1962; Agosta 2002; Lacki et al. 2009). Given we found no differences in wavelength (and therefore color of light emitted) correlating to these roosting preferences, we suggest that the color of photoluminescence is not likely to be specifically niche‐adapted for the roosting environment in these species.

While echolocation is regarded as the primary sensory modality in bats, vision also plays an important role. Members of the family Pteropodidae rely primarily on vision as opposed to laryngeal echolocation (Graydon et al. 1987). Medium to longwave sensitive (M/LWS) vision is highly conserved in bats, with M/LWS opsins able to absorb light in the mid‐500 nm range (Wang et al. 2004; Zhao et al. 2009; Simões et al. 2019). Simões et al. (2019) found the M/LWS opsin to be maximally sensitive to a range of 536–560 nm, coinciding with the peak ranges observed in the species we examined. Therefore, detection of the wavelengths of photoluminescent emission we documented is likely; however, we did not explicitly investigate M/LWS vision in the species we examined.

Quantifying the wavelength range of photoluminescence and comparison with optical sensitivity ranges provides support for a potential ecological function, but there are additional factors to be considered. While bats are active in nocturnal and twilight hours when ultraviolet and low‐wavelength blue light is abundant, it is not known whether the amount of excitation light present in the environment is sufficient to produce the photoluminescent emission we observed, especially in dark roosting environments. In addition, the emission we quantified was present on the ventral uropatagium and limbs, locations that may be visible during flight and foraging but less so during roosting.

Evaluation of behavioral relevance of photoluminescence is another important factor to consider when examining functional significance. Bats differ from many of the other nocturnal mammal groups in which photoluminescence has been observed in the widespread complexity of social structures they exhibit (Kerth 2008; Wilkinson et al. 2019). Several of the species we examined, including the *Myotis* species, *E. fuscus* , and *T. brasiliensis* , form social aggregations (Rice 1957; Davis et al. 1962; Phillips 1966), which would provide opportunities for photoluminescence to serve a function in communication. However, studies evaluating a difference in behavior in the presence and absence of photoluminescence would be required to confirm whether a photoluminescent signal has any social significance (Marshall and Johnsen 2017). Our results suggest a shared physiological origin, but we cannot confirm any shared behavioral function.

We recognized the potential for older specimens to exhibit differences in photoluminescence characteristics as a result of degradation. However, our results did not support this supposition. The lesions caused by *Pseudogymnoascus destructans* in bats with white‐nose syndrome are photoluminescent under ultraviolet light (Turner et al. 2014), and bacteria within the *Pseudomonas* genus common in the cutaneous microbiome of bats (Avena et al. 2016; Hoyt et al. 2015) are known to fluoresce green (Meyer and Abdallah 1978). The presence of these organisms could potentially confound the wavelengths detected by spectroradiometer scans; however, they are unlikely to be present in preserved specimens. By examining study skins, we were able to minimize confounding biological sources of photoluminescence and isolate wavelengths originating from specimens. While we found no influence of specimen age and did not observe the presence of other factors influencing wavelength values, comparison of photoluminescence in live individuals with museum specimens would be important to confirm characteristics and further investigate potential ecological function. Additionally, all specimens examined were adults at the time of collection. Comparative research on characteristics of photoluminescence between juveniles and adults could offer additional insights into the ontogeny of the trait.

The term photoluminescence encompasses several mechanisms by which light is emitted following initial excitation, including true fluorescence and light scattering (Clarke and Oprysa 2004). Studies incorporating multiple mammal taxa by Toussaint et al. (2023) and Travouillon et al. (2023) confirmed emission was a result of fluorescence as opposed to light scattering. Further tests involving excitation at multiple wavelengths would be required to conclude the same for the species studied here. Whether light emission is due to true fluorescence or light scattering is not likely to be a significant factor influencing ecological function. However, additional distinction of mechanisms responsible for photoluminescence may provide greater insight into the molecular origins of emission.

Quantifying characteristics of emission allowed us to develop hypotheses regarding phylogenetic patterns and potential adaptive significance of photoluminescence. Our results suggest that photoluminescence is homologous in origin for the species we examined and is not likely to function in sexual selection or camouflage. Due to the quality of data produced by specimens of a range of ages, specimen collections are an important source of data for photoluminescence studies, and we emphasize the value of maintaining comprehensive specimen collections to inform future research. We suggest that future studies quantify differences in photoluminescent characteristics between specimens and live individuals, and that further explicit testing of the potential ecological functions of photoluminescence relating to differences in life history would be beneficial.

## Author Contributions

**Briana J. Roberson:** conceptualization (lead), data curation (lead), formal analysis (equal), investigation (lead), methodology (lead), writing – original draft (lead). **Santiago Perea:** conceptualization (supporting), formal analysis (equal), supervision (lead), writing – review and editing (equal). **Daniel DeRose‐Broeckert:** conceptualization (supporting), investigation (supporting), methodology (supporting), writing – review and editing (supporting). **Steven B. Castleberry:** conceptualization (supporting), project administration (lead), resources (lead), supervision (supporting), writing – review and editing (supporting).

## Conflicts of Interest

The authors declare no conflicts of interest.

## Supporting information


Table S1.

## Data Availability

R code and museum specimen information are uploaded as Supporting Information. We will provide raw data from spectral scans on Dryad. We attempted to upload the raw data as Supporting Information but the file was too large to upload.
